# Chronotropic Intolerance: An Overlooked Determinant of Symptoms and Activity Limitation in Myalgic Encephalomyelitis/Chronic Fatigue Syndrome?

**DOI:** 10.3389/fped.2019.00082

**Published:** 2019-03-22

**Authors:** Todd E. Davenport, Mary Lehnen, Staci R. Stevens, J. Mark VanNess, Jared Stevens, Christopher R. Snell

**Affiliations:** ^1^Department of Physical Therapy, Thomas J. Long School of Pharmacy and Health Sciences, University of the Pacific, Stockton, CA, United States; ^2^Workwell Foundation, Ripon, CA, United States; ^3^Department of Health, Exercise, and Sport Sciences, College of the Pacific, University of the Pacific, Stockton, CA, United States

**Keywords:** myalgic encephalomyelitis (ME), exercise, exercise test, heart rate, chronotropic incompetence (CI), chronic fatigue syndrome

## Abstract

Post-exertional malaise (PEM) is the hallmark clinical feature of myalgic encephalomyelitis/chronic fatigue syndrome (ME/CFS). PEM involves a constellation of substantially disabling signs and symptoms that occur in response to physical, mental, emotional, and spiritual over-exertion. Because PEM occurs in response to over-exertion, physiological measurements obtained during standardized exertional paradigms hold promise to contribute greatly to our understanding of the cardiovascular, pulmonary, and metabolic states underlying PEM. In turn, information from standardized exertional paradigms can inform patho-etiologic studies and analeptic management strategies in people with ME/CFS. Several studies have been published that describe physiologic responses to exercise in people with ME/CFS, using maximal cardiopulmonary testing (CPET) as a standardized physiologic stressor. In both non-disabled people and people with a wide range of health conditions, the relationship between exercise heart rate (HR) and exercise workload during maximal CPET are repeatable and demonstrate a positive linear relationship. However, smaller or reduced increases in heart rate during CPET are consistently observed in ME/CFS. This blunted rise in heart rate is called chronotropic intolerance (CI). CI reflects an inability to appropriately increase cardiac output because of smaller than expected increases in heart rate. The purposes of this review are to (1) define CI and discuss its applications to clinical populations; (2) summarize existing data regarding heart rate responses to exercise obtained during maximal CPET in people with ME/CFS that have been published in the peer-reviewed literature through systematic review and meta-analysis; and (3) discuss how trends related to CI in ME/CFS observed in the literature should influence future patho-etiological research designs and clinical practice.

## Introduction

Myalgic encephalomyelitis/chronic fatigue syndrome (ME/CFS) is estimated to affect 0.8 to 2.5 million people in the United States (1). Ninety percent of cases are thought to go undiagnosed (1), suggesting that people with ME/CFS are substantially under-counted, under-diagnosed, and under-treated. The hallmark clinical feature of ME/CFS is post-exertional malaise (PEM), which involves a constellation of substantially disabling signs and symptoms that occur in response to physical, mental, emotional, and spiritual over-exertion. A number of criteria for ME/CFS exist for clinical and research purposes (1–5). Criteria including PEM appear to have the best face validity to differentiate ME/CFS from other fatiguing health conditions (1, 6, 7). The pervasive nature of PEM in ME/CFS has led some working groups to revise diagnostic criteria for ME/CFS to highlight the multi-system deficits associated with exertion intolerance (1–3).

The importance of PEM in ME/CFS emphasizes the value of studies that document abnormalities in exercise response to advance understanding of the patho-etiology, potential biomarkers, and functional disability associated with ME/CFS. Heart rate is one objective measurement, which can be reliably obtained from wearable biometric technology. A large body of literature already exists that documents heart rate responses to exercise in ME/CFS and other fatiguing health conditions. The increasing availability and affordability of wearable biometric technology has led to the observation that wearables could be used for activity tracking and prediction of PEM, using cardiac function as an early proxy for future symptoms. Therefore, the purposes of this perspective are to (1) review the mechanisms for cardiac control during exercise; (2) review the literature related to heart rate responses and exercise in ME/CFS; and (3) discuss the potential implications for aberrant heart rate responses in ME/CFS and its relationship to interpreting the results of exercise testing paradigms and analeptic activity management.

## The Relationship Between Heart Rate and Workload is Repeatable and Predictable

Under normal conditions, the relationship between heart rate and workload increases linearly. Reliability of a measure is a precursor to validity. Exercise heart rates at maximal exertion and ventilatory anaerobic threshold (VAT) are highly reproducible in both non-disabled individuals and individuals with various health conditions (8–19). In addition, the relationship between workload and heart rate is normally very reproducible (20). That is to say, the correlation is subject to very low error variance. These observations suggest that deviations in the incremental increase in heart rate in response to each unit increase in workload might suggest pathology. In other words, variation in measurements during cardiopulmonary exercise testing (CPET) in people with ME/CFS may reflect true biological variance that can be functionally relevant and provide important patho-etiological clues about the nature of ME/CFS. In healthy people, peak VO_2_ reflects a 4-fold increase over resting VO_2_ (21), which is accomplished by a 2.2-time increase in heart rate, a 0.3-fold increase in stroke volume, and a 1.5-fold increase in arteriovenous oxygen difference (21). The elevation of one's heart rate is the largest contributor to both VO_2_ and the ability to maintain exercise at maximal level workloads (21). Further, an increase in heart rate is a variable of great interest to clinicians and researchers when observing abnormal responses to exertion and predicting possible consequences due to those abnormal responses. A normal and intact heart rate response pattern to exertion is necessary because cardiac output (heart rate × stroke volume) must be matched to metabolic demands throughout the duration of exercise.

## Impairment in Chronotropic Response is Measurable

Chronotropic intolerance (CI) is defined by a range of different criteria, including; failure to achieve age-predicted maximal heart rate, delays in achieving age-predicted maximal heart rate, inadequate heart rates at submaximal workloads, slowed post-exertion recovery heart rate, or heart rate fluctuations (21, 22). The prevalence of CI is poorly understood because it is non-uniformly defined. Gentlesk et al. (22) reported the prevalence of CI ranges from 3.1 to 11% in patients referred for exercise testing, >40% in a population of patients with pacemakers, and up to 60% in patients with atrial fibrillation (22). This variation in prevalence provides further evidence in support of the need for a clear definition and a standardized set of criteria so that diagnosis of CI may be made appropriately and populations can be compared (21).

CI is most often diagnosed using a percentage as the cutoff for distinguishing between normal and abnormal heart rate responses to incremental increases in workload during an exercise test (23). The most common percentages of age-predicted maximal heart rate that have been used range between 70 and 85% (23). CI also can be represented as a measure of heart rate reserve, which is the change in heart rate from rest to peak exercise measured during an exercise test (23). However, since the heart rate reserve equation is dependent upon the resting heart rate, it can be taken one step further to better represent an individual's heart rate response to exercise (23). In other words, chronotropic response can be calculated as a fraction of heart rate reserve achieved at maximal effort, given by $\frac{\left|\Delta HR_{rest\to peak}\right|}{\left(220-age\right)-HR_{peak}}$ (23). Failure to obtain ≥80% of the adjusted heart rate reserve during an incremental exercise test is the most common criterion used to distinguish CI (23). Some researchers prefer to take a more definitive route when measuring exertion. The ratio of the volume of carbon dioxide produced to the volume of oxygen consumed, or the respiratory exchange ratio, represents an objective measure of physiologic effort during exertion (23). It is generally accepted that a respiratory exchange ratio of >1.15 is indicative of intense, maximal exercise, while a ratio of <0.82 is indicative of a resting state. If an individual's respiratory exchange ratio is <1.05 at peak exercise, research suggests that this indicates either a submaximal level of effort or a premature termination of the exercise test and should be analyzed with caution (23). Similarly, in 1992, Wilkoff et al. (24) attempted to diagnose CI in a more objective manner through the use of the metabolic-chronotropic relationship, or the chronotropic index, which is the ratio between heart rate reserve and metabolic reserve during submaximal workloads. Wilkoff et al. (24) chose this method because it adjusts for age, physical fitness level, functional capacity, and it is unaffected by a researcher's choice of exercise test or protocol. Under normal conditions in healthy individuals, the percentage of heart rate reserve should match the percentage of metabolic reserve achieved during exertion to equal a chronotropic index of 1.0 with 95% confidence intervals of 0.8 and 1.3 (24). Therefore, if the metabolic-chronotropic relationship, or chronotropic index, is ≤0.8 from a given slope or single value throughout one stage of an incremental exercise test, then that is considered CI (24). The Wilkoff model for CI is given as HR_stage_ = $\frac{\left[\left(220-age\right)-HR_{rest}\right]\;*\;\left(METs_{stage}-1\right)}{\left(METS_{peak}-1\right)\;+HR_{rest}}$, and depends on age, resting heart rate, age-predicted maximal heart rate, age-predicted heart rate reserve, maximal heart rate observed during exercise testing, volume of oxygen consumed (VO_2_–expressed as MET values; 3.5 ml/kg/min) at each stage and at peak exertion, and respiratory exchange ratio (24). Further, this equation can be combined with the previously discussed methods of age-predicted maximal heart rate, adjusted heart rate reserve, and respiratory exchange ratio to determine whether or not CI is present. For example, chronotropic index can be used as a deciding factor if a subject achieves an adequate peak respiratory exchange ratio of >1.09, but fails to achieve ≥80 or 85% of adjusted heart rate reserve or age-predicted maximal heart rate, or if a subject achieves a peak respiratory exchange ratio of <1.09 (21). One can see that there are a number of methods for distinguishing between a normal chronotropic response and CI, which is dependent upon a handful of variables. It is imperative that researchers work together to create a definition and criteria that are clearly defined to consistently identify CI.

## Fatiguing Health Conditions Involve Impaired Chronotropic Responses

Lauer et al. (25) examined prognostic implications of CI in 1,575 asymptomatic male participants from the Framingham Offspring Study. In order to be designated asymptomatic, participants were required to take part in an exercise treadmill test (25). Researchers followed the participants for an average of 7.7 years to investigate all-cause mortality and coronary heart disease events, including angina pectoris, coronary insufficiency, myocardial infarction, any type of coronary heart disease deaths, and coronary revascularization (25). The treadmill exercise test was terminated when participants achieved 85% of age- and sex-predicted maximal heart rate (25). Lauer et al. (25) also mentioned that treadmill tests were terminated upon “participant request, limiting chest discomfort, dyspnea, fatigue, leg discomfort, hypotension, an excessive increase in systolic blood pressure (i.e., peak systolic pressure ≥250 mmHg), ≥2 mm ST-segment depression, or significant ventricular ectopy. Researchers distinguished between normal and abnormal chronotropic responses using three different variables—the ability or inability to achieve 85% of his age- and sex-predicted maximal heart rate, an increase in heart rate from rest to peak, and the chronotropic index at stage 2 of the Bruce protocol (25). One thousand two hundred and forty-eight participants (79%) achieved 85% of their age-predicted maximal heart rates, while the remaining 327 participants (21%) failed to achieve 85% of the target heart rate (25). The participants that failed to reach the target heart rate were also at an increased risk for an ischemic ST-segment response to appear on an ECG, had a lower exercise capacity, and were related to higher occurrences of all-cause mortality and coronary heart disease events (25). The researchers found that increases in heart rate with exertion were inversely related to mortality risk and that an impaired chronotropic response index was also predictive of mortality (25).

## Empirical Data Suggest Chronotropic Impairment is Present in People With ME/CFS

Our group (26, 27) and others (28–30) have measured heart rate responses to exercise in ME/CFS using CPET methodology that allows for careful characterization at peak exertion and VAT. The specific protocol our group has used for over 20 years was developed to capture the difference in underlying physiology between the average symptomatic state and potential cardiovascular, pulmonary, and metabolic decrements characteristic of PEM (26, 28, 31–33). To begin, patients are instructed to rest as much as possible before performing the first CPET, which measures a baseline of the individual and provides a physical stressor to induce PEM. A second CPET performed 24 h after the first is then conducted to measure the individual's response to exercise while in a post exertional state. Sedentary but otherwise non-disabled individuals exhibit high levels of reproducibility between tests (19, 34). Even individuals with various health conditions that present with fatigue demonstrate reproducible CPET measurements (9, 10, 13–17). However, the physiological correlates of PEM, which are typically exacerbated by exertion, are often indicated by variation outside expected intervals in successive exercise tests. Therefore, changes on the test are not related to poor reliability (i.e., “error variance”), but rather the biological variance associated with ME/CFS.

**Figure 1:**
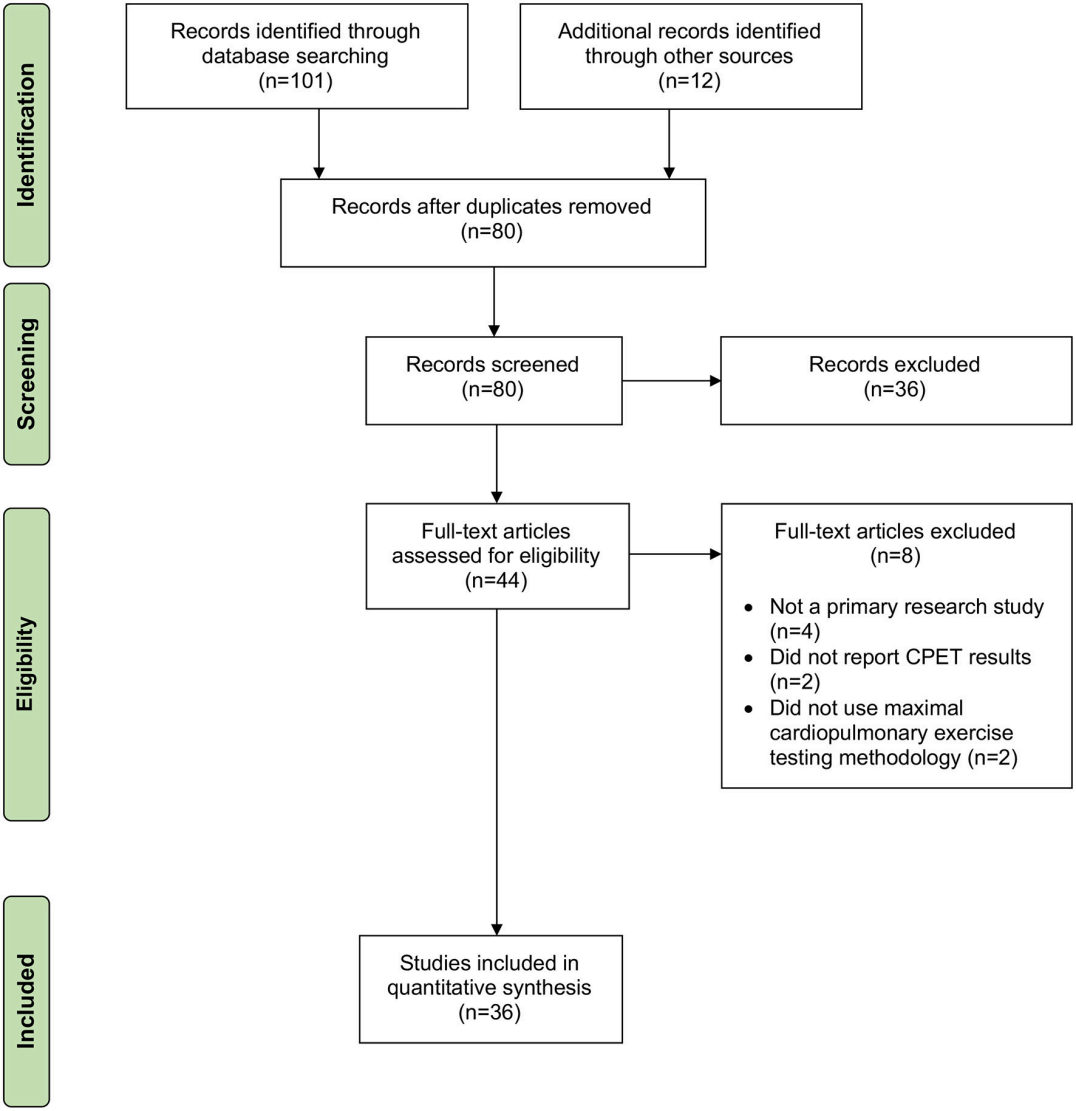
Flow diagram describing the systematic review.

We conducted a systematic review to locate primary research articles published in the peer reviewed and so-called unpublished “gray literature” that described chronotropic responses to exercise during maximal cardiopulmonary exercise testing in people with ME/CFS, with or without comparison to matched control subjects. Maximal cardiopulmonary exercise testing was chosen because there are uniform criteria described for test cessation, and documented criteria exist to identify physiological performance at the ventilatory anaerobic threshold (VAT), which is the point at which non-oxidative or anaerobic metabolism begins to significantly contribute to energy metabolism with increasing exercise workloads (35, 36). Articles that reported mean age of participants and heart rate at either peak exertion or VAT were included in the quantitative analysis. We searched Medline Complete, CINAHL, Academic Search Complete, SPORTDISCUS, and PsycINFO on 5 December 2018 using keywords [*(SU exercise tests) OR (exercise physiology) OR (cardiopulmonary system)*] *AND* [*(SU myalgic encephalomyelitis) OR (SU chronic fatigue syndrome)*]. We also conducted hand searches of reference sections and included other known papers that were not included in the search results. The systematic review revealed 36 articles that were included in the quantitative analysis (Figure 1).

CPET responses on a single test were assessed in the context of a single maximal CPET in patients with ME/CFS only (14 studies, including 1,169 patients with ME/CFS) compared with otherwise non-disabled individuals who were matched for gender and age (17 studies, including 961 patients with ME/CFS and 529 control subjects; Tables 1–**3**). Among these studies, 25 studies (28–30, 37–42, 47, 48, 52–60, 62, 63, 65–69) used the Fukuda et al. criteria (4), four studies (43–45, 51) used the Oxford criteria (5), five studies used the Holmes criteria (49, 50, 61, 64, 70), and one study (46) used the Fukuda et al. criteria, Canadian Consensus Criteria (2), and International Consensus Criteria (3). An additional four studies (30, 66) compared measurements obtained during a single CPET between men and women with ME/CFS (**Table 3**); three studies used the Fukuda criteria to identify ME/CFS (4). Three other studies (28, 46, 65) compared the responses of individuals with ME/CFS on two CPETs spaced 24 h apart. Two of the studies (28, 65) used the Fukuda et al. criteria (4) and one study (46) used a combination of the Fukuda et al. criteria (4), Canadian Consensus Criteria (2), and International Consensus Criteria (3). Raw HR data were extracted from each study at maximal exertion and VAT, as available. Age-predicted maximum HR values were calculated as 220−*meanage*_*sample*_. Predicted VAT HR values were taken as 70% of predicted maximum HR (71, 72). Percentage of age-predicted maximum heart rate was computed by dividing the observed exercise heart rate by its respective age-predicted value.

**Table 1:** Heart rate measurements obtained at peak exertion during a single maximal cardiopulmonary exercise test in studies comparing subjects with myalgic encephalomyelitis/chronic fatigue syndrome (*n* = 2,270) to matched control subjects (*n* = 594).

**Study**	**Case definition criteria**	**Control subjects**				**Patients with ME/CFS**			
		***n***	**Observed**	**Predicted**	**% Predicted**	***n***	**Observed**	**Predicted**	**% Predicted**
**HEART RATE AT PEAK EXERTION**									
Bazelmans et al. (37)	Fukuda	20	173	186	93.0	20	165	187	88.2
Blazquez et al. (38)	Fukuda	—	—	—	—	32	129	180	71.7
Castro-Marrero et al. (39)	Fukuda	—	—	—	—	73	140	171	81.9
Cook et al. (40)	Fukuda	20	183	187	97.9	19	174	186	93.6
Cook et al. (41)	Fukuda	19	163	177	92.1	15	156	178	87.6
Cook et al. (42)	Fukuda	32	173	183	94.5	29	169	180	94.0
De Becker et al. (29)	Fukuda	204	171	184	92.9	427	151	183	82.5
Fulcher and White (43)	Oxford	30	182	183	99.5	66	171	183	93.4
Gallagher et al. (44)	Oxford	42	183	185	98.9	41	178	182	97.8
Gibson et al. (45)	Oxford	12	190	188	101.1	12	163	187	87.2
Hodges et al. (46)	Fukuda, CCC, ICC	10	161	181	89.0	10	154	183	84.2
Ickmans et al. (47)	Fukuda	13	165	191	86.4	30	145	184	78.8
Inbar et al. (48)	Fukuda	15	172	177	97.2	15	150	177	84.8
Keller et al. (28)	Fukuda	—	—	—	—	22	159	176	90.3
Kent-Braun et al. (49)	Holmes	—	—	—	—	6	—	—	93.0
Montague et al., (50)	Holmes	41	152	184	82.6	41	124	184	67.4
Mullis et al. (51)	Oxford	—	—	—	—	130	140	181	77.4
Nagelkirk et al. (52)	Fukuda	19	163	177	92.1	15	156	178	87.6
Nijs et al. (53)	Fukuda	—	—	—	—	64	140	180	77.8
Nijs et al. (54)	Fukuda	—	—	—	—	240	144	186	77.4
Nijs et al. (55)	Fukuda	—	—	—	—	77	140	179	78.2
Nijs et al. (56)	Fukuda	—	—	—	—	28	146	178	82.0
Nijs et al. (57)	Fukuda	—	—	—	—	16	159	182	87.4
Nijs et al. (58)	Fukuda	—	—	—	—	156	152	181	84.0
Nijs et al. (59)	Fukuda	—	—	—	—	36	146	181	80.7
Pardaens et al. (60)	Fukuda	—	—	—	—	116	142	181	78.5
Riley et al. (61)	Holmes	13	182	186	97.9	13	177	186	95.2
Robinson et al. (62)	Fukuda	6	173	176	98.3	6	177	175	101.1
Sargent et al. (30)	Fukuda	33	186	185	100.5	33	184	186	98.9
Shukla et al. (63)	Fukuda	10	179	173	103.5	10	159	171	93.0
Sisto et al. (64)	Holmes	22	178	187	95.2	21	161	186	86.6
Van Ness et al. (27)	Holmes	—	—	—	—	179	140	177	79.1
Vermeulen et al. (65)	Fukuda	15	167	184	90.7	15	158	184	86.4
Vermeulen and Vermeulen van Eck (66)	Fukuda	18	159	175	90.8	223	158	182	85.9
Sample weighted mean		—	172.3	183.4	94.0	—	149.8	181.4	82.2
95% confidence interval		—	171.3–173.3	182.9–183.8	93.6-94.4	—	149.2–150.4	181.3–181.6	81.9–82.5

*n, sample size; ME/CFS, myalgic encephalomyelitis/chronic fatigue syndrome; CCC, Canadian Consensus Criteria; ICC, International Consensus Criteria*.

**Figure 2:**
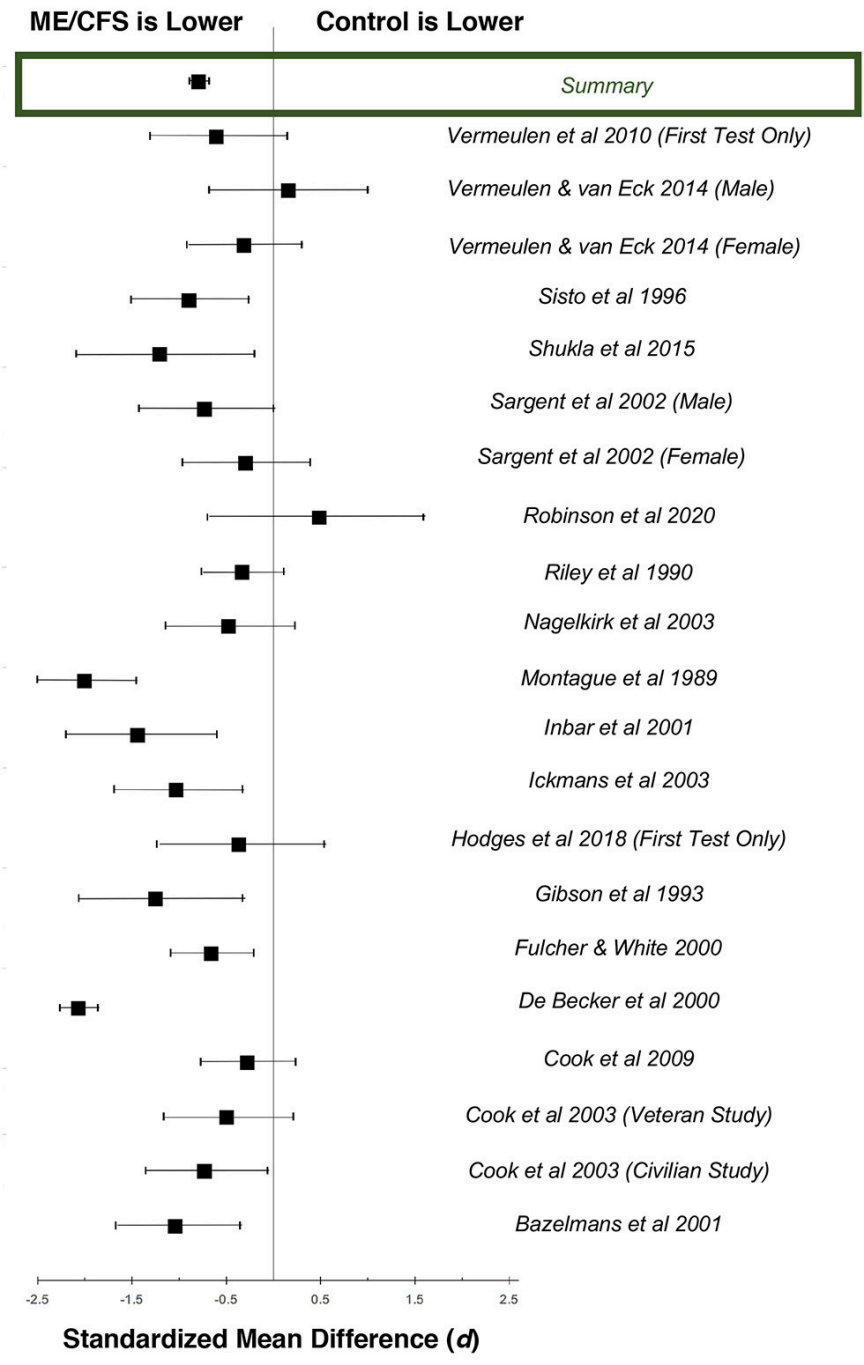
Standardized mean differences (*d*) for heart rate at peak exertion during maximal cardiopulmonary exercise testing, comparing patients with ME/CFS (*n* = 1,053) and matched control subjects (*n* = 569). Boxes represent point estimates, and whiskers are 95% confidence intervals. Patients with ME/CFS had lower peak heart rates than matched control subjects (large effect size).

**Figure 3:**
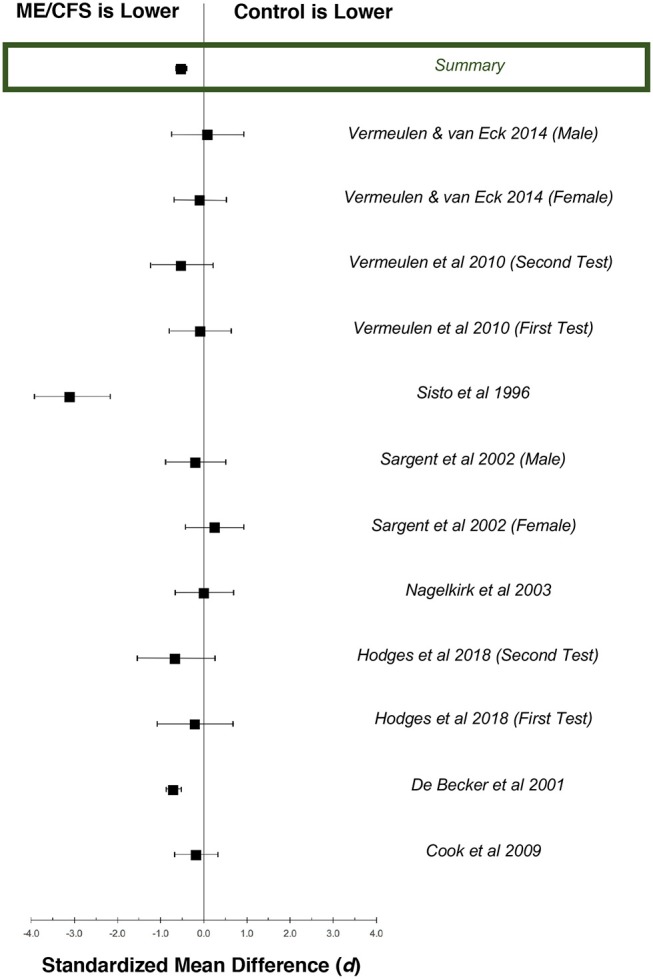
Standardized mean differences (*d*) for exercise heart rate at ventilatory anaerobic threshold (VAT), comparing patients with ME/CFS (*n* = 778) and matched control subjects (*n* = 378). Boxes represent point estimates, and whiskers are 95% confidence intervals. Patients with ME/CFS had lower heart rates at VAT than matched control subjects (moderate effect size).

Data from each study were pooled by calculating sample-weighted mean values for HR and 95% confidence interval (ConI) from the relevant studies, in order to conduct the following assessments: (1) to compare chronotropic responses to exercise in individuals with ME/CFS compared to matched control subjects, (2) to evaluate the effect of gender on HR responses to activity in individuals with ME/CFS, (3) to determine the effect of serial CPET on chronotropic response in individuals with ME/CFS, and (4) to estimate the effect of cardiovascular impairment on chronotropic response in individuals with ME/CFS. In addition, standardized mean difference and 95% ConI were calculated from studies that compared ME/CFS to matched control subjects, in order to estimate the magnitude of effect (73). A variance weighted summary also was calculated to pool the results across all studies. These results were used to generate forest plots for the data at peak exertion (Figure 2) and ventilatory anaerobic threshold (Figure 3). Q and *I*^2^ statistics were assessed to determine the amount of statistical heterogeneity across studies (74). Pooled standard deviations were computed using a random effects model. Point estimates for pooled data were compared using independent samples *t*-tests. All analyses were considered statistically significant at *p* < 0.05.

### Comparisons Between Patients With ME/CFS and Matched Control Subjects

There were 36 studies that reported heart rate responses at peak exertion in individuals with ME/CFS (*n* = 2,270) and 21 studies involving matched control subjects (*n* = 594; Table 1). Control subjects performed at 94.0% of age-predicted maximum HR (95%ConI: 93.6–94.4%), while individuals with ME/CFS performed at 82.2% (81.9–82.5%) of age-predicted maximum HR (*p* < 0.0001). Almost all the studies measured a decreased peak HR in individuals with ME/CFS. The standardized mean difference (*d*) for these data was −1.37 (95%ConI: −1.46 to −1.26), which indicates a very large effect, and 92% of the ME/CFS group had a peak exercise heart rate that was below the matched control group. This corresponded to an unstandardized mean difference of 11.2 fewer beats per minutes in patients with ME/CFS compared to matched control subjects (95%ConI: 6.9–15.4 bpm decrease). Significant heterogeneity was present in available studies (*Q* = 113.8, *p* < 0.0001; *I*^2^: 82%), so these pooled difference estimates should be viewed with caution. Despite the heterogeneity present in this literature for each pooled effect size estimate, the high number of included studies and pooled sample size provides for substantial statistical power. Potential sources of variability in the published literature include the differences in case definitions used for ME/CFS, fitness levels of matched control subjects relative to patients with ME/CFS, testing modality (i.e., treadmill vs. bicycle), and statistical noise introduced by reliability of criteria to select peak performance between studies. Despite these methodological differences, published data indicate the presence of statistically significant and clinically relevant impairment in chronotropic response to exercise at peak exertion in individuals with ME/CFS compared to matched control subjects.

Twelve datasets from nine studies documented chronotropic responses at VAT in individuals with ME/CFS (*n* = 795) compared to control subjects (*n* = 353; Table 2). Overall, control subjects performed at 107.0% (95%ConI: 105.9–108.1%) and individuals with ME/CFS performed at 97.9% (95%ConI: 97.4–98.4%) of their age-predicted heart rates (*p* < 0.0001). This finding indicates patients with ME/CFS, on average, remained relatively impaired when compared to age- and sex-matched control subjects. Seven of nine studies documenting chronotropic responses at VAT showed a decrease in patients with ME/CFS compared to matched control subjects, while the remaining two studies found slight increases. Overall, the standardized mean difference (*d*) for these data was −0.53 (95%ConI: −0.65 to −0.40), which indicates a moderate effect. Sixty-three percent of patients with ME/CFS had lower heart rates at VAT than matched controls in the context of a single test. These findings correspond to an unstandardized mean difference of 5.4 fewer beats per minutes in patients with ME/CFS compared to matched control subjects (95%ConI: 1.5–9.2 bpm decrease). Moderate heterogeneity was present in available studies (*Q* = 30.01, *p* < 0.01; *I*^2^ = 60%). Like the peak exercise analysis, the relatively high pooled sample size provides substantial statistical power. However, it is notable that data evaluating heart rate at VAT from De Becker et al. (29) and Vermeulen and Vermeulen van Eck (66) differ by over 20 percentage points in people with ME/CFS (105.1 and 85.6%, respectively), and exert a large influence on sample-weighted means for observed heart rate and percent of predicted heart rate due to large sample sizes (*n* = 427 and *n* = 204, respectively). This observation highlights the need to consider the unique physiological characteristics of individual patients with ME/CFS. Some of the observed variation also may be attributed to heterogeneous methods used to select VAT used in the literature, indicating the need to identify and observe uniform methods of CPET analysis (75).

**Table 2:** Heart rate measurements obtained at ventilatory anaerobic threshold during a single maximal cardiopulmonary exercise test in studies comparing subjects with myalgic encephalomyelitis/chronic fatigue syndrome (*n* = 795) to matched control subjects (*n* = 353).

**Study**	**Case definition criteria**	**Control subjects**				**Patients with ME/CFS**			
		***n***	**Observed**	**Predicted**	**% Predicted**	***n***	**Observed**	**Predicted**	**% Predicted**
**HEART RATE AT VENTILATORY ANAEROBIC THRESHOLD**									
Cook et al. (42)	Fukuda	32	112	128	87.4	29	109	126	86.5
De Becker et al. (29)	Fukuda	204	150	129	116.5	427	135	128	105.4
Hodges et al. (46)	Fukuda, CCC, ICC	10	137	127	108.1	10	134	128	104.6
Keller et al. (28)	Fukuda	—	—	—	—	22	114	123	92.5
Nagelkirk et al. (52)	Fukuda	19	110	124	88.7	15	111	125	88.8
Sargent et al. (30)	Fukuda	33	126	130	97.2	33	127	130	97.5
Sisto et al. (64)	Holmes	22	130	131	99.3	21	119	130	91.4
Vermeulen et al. (65)	Fukuda	15	111	129	97.7	15	110	129	85.6
Vermeulen and Vermeulen van Eck (66)	Fukuda	18	109	111	84.0	223	112	128	82.9
Sample weighted mean		—	136.8	116.1	107.0	—	125.2	127.9	97.9
95% confidence interval		—	135.1–138.4	115.4–116.8	105.9–108.1	—	124.5-125.9	127.7-128.0	97.4-98.4

*n, sample size; ME/CFS, myalgic encephalomyelitis/chronic fatigue syndrome; CCC, Canadian Consensus Criteria; ICC, International Consensus Criteria*.

### Comparisons Between Females and Males With ME/CFS

Articles describing two studies of CPET measurements in individuals with ME/CFS permitted abstraction of data by subject sex (30, 66), involving 1,104 females and 58 males with measurements at peak exertion and 41 males and 195 females with measurements at VAT (Table 3). Males demonstrated a significantly higher achievement of age-predicted maximum heart rate at peak exertion (females: 83.0%, 95%ConI: 82.6–83.4%; males: 87.5%, 95%ConI: 85.4–89.7%; *p* < 0.0001) but not VAT (females: 88.6%, 95%ConI: 87.3–89.9%; males: 87.5%, 95%ConI: 85.2–89.7%; *p* = 0.476). These data suggest that, although there may be important sex-related features in ME/CFS incidence, the expression of CI in ME/CFS appears homogeneous between sexes at submaximal workloads (75). Additional studies of sex-related difference in CI at peak levels of exertion are warranted, because male patients with ME/CFS appear under-represented in the literature to date.

**Table 3:** Heart rate measurements obtained at peak exertion and ventilatory anaerobic threshold during a single maximal cardiopulmonary exercise test in studies comparing females (*n* = 1,104) and males (*n* = 58) with myalgic encephalomyelitis/chronic fatigue syndrome.

**Study**	**Case definition criteria**	**Females with ME/CFS**				**Males with ME/CFS**			
		***n***	**Observed**	**Predicted**	**% Predicted**	***n***	**Observed**	**Predicted**	**% Predicted**
**HEART RATE AT PEAK EXERTION**									
Blazquez et al. (38)	Fukuda	32	126	187	71.7	—	—	—	—
Castro-Marrero et al. (39)	Fukuda	73	140	171	81.8	—	—	—	—
Cook et al. (40)	Fukuda	19	174	186	93.6	—	—	—	—
De Becker et al. (29)	Fukuda	427	151	183	82.5	—	—	—	—
Ickmans et al. (47)	Fukuda	30	145	184	78.8	—	—	—	—
Montague et al. (50)	Unknown	20	126	187	67.4	11	119	180	66.1
Nijs et al. (58)	Fukuda	156	152	181	84.0	—	—	—	—
Nijs et al. (59)	Fukuda	36	146	181	80.7	—	—	—	—
Pardaens et al. (60)	Fukuda	116	142	181	78.5	—	—	—	—
Robinson et al. (62)	Fukuda	—	—	—	—	6	173	176	98.3
Sargent et al. (30)	Fukuda	17	183	186	98.4	16	184	186	98.9
Vermeulen and Vermeulen van Eck (66) (CFS Only)	Fukuda	178	158	177	89.3	25	155	178	87.0
Sample weighted mean		—	150.1	180.8	83.0	—	158.0	180.4	87.5
95% confidence interval		—	149.0–151.1	180.4–181.2	82.6–83.4	—	152.8–163.3	178.9–181.8	85.4–89.7
**HEART RATE AT VENTILATORY ANAEROBIC THRESHOLD**									
Sargent et al. (30)	Fukuda	17	131	130	100.6	16	122	130	93.7
Vermeulen and Vermeulen van Eck (66) (CFS Only)	Fukuda	178	112	128	87.4	25	104	125	83.5
Sample weighted mean		—	113.7	128.3	88.6	—	110.0	126.8	87.5
95% confidence interval		—	111.8–115.5	128.1–128.5	87.3–89.9	—	107.1–114.9	125.6–128.0	85.2–89.7

*N, sample size; ME/CFS, myalgic encephalomyelitis/chronic fatigue syndrome*.

### Comparisons Between Measurements Obtained During Serial CPETs

There were three studies involving two CPETs conducted 24 h apart (28, 46, 65), comprising 47 patients with ME/CFS and 35 matched control subjects (Table 4). On the first CPET at maximal exertion, individuals with ME/CFS demonstrated a significantly lower heart rate response that was 87.9% of predicted by age (95%CI: 86.9–88.9%) compared to control subjects with a heart rate response of 90.0% of predicted by age (95%ConI: 89.5–90.5%; *p* < 0.01). On the second CPET at peak exertion, control subjects maintain the heart rate response to exercise compared to age-predicted norms (90.6%; 95%ConI: 90.1–91.1%) but individuals with ME/CFS demonstrated a significant decline compared to control subjects (84.3%; 95%ConI: 83.9–84.7%; *p* < 0.05). Although peak exertion is not common in daily life, sympathetic autonomic drive is maximal during peak exertion, so this observed difference may magnify subtle decrements in sympathetic autonomic drive that may only inconsistently be observed during lower levels of physical exertion.

**Table 4:** Heart rate measurements obtained at peak exertion and ventilatory anaerobic threshold during studies involving two cardiopulmonary exercise tests in individuals with myalgic encephalomyelitis/chronic fatigue syndrome (*n* = 47) and matched control subjects (*n* = 35).

**Study**	**Case definition criteria**	**Test 1**				**Test 2**			
		***n***	**Observed**	**Predicted**	**% Predicted**	***n***	**Observed**	**Predicted**	**% Predicted**
**HEART RATE AT PEAK EXERTION IN PATIENTS WITH ME/CFS**									
Hodges et al. (46)	Fukuda, CCC, ICC	10	154	183	84.2	10	151	183	82.5
Keller et al. (28)	Fukuda	22	160	176	90.9	22	150	176	85.2
Vermeulen et al. (65)	Fukuda	15	158	184	85.7	15	155	184	84.2
Sample weighted mean		—	158.1	180.0	87.9	—	151.8	180.0	84.3
95% confidence interval		—	157.2–159.0	178.8–181.3	86.9–88.9	—	151.1–152.6	178.8–181.3	83.9–84.7
**MEASUREMENTS AT PEAK EXERTION IN CONTROL SUBJECTS**									
Hodges et al. (46)	Fukuda, CCC, ICC	10	161	181	89.0	10	162	181	89.5
Vermeulen et al. (65)	Fukuda	15	167	184	90.8	15	168	184	91.3
Sample weighted mean		—	164.6	182.8	90.0	—	165.6	182.8	90.6
95% confidence interval		—	162.9–166.3	182.0–183.6	89.5–90.5	—	163.9–167.6	182.0–183.6	88.1–93.6
**HEART RATE AT VENTILATORY ANAEROBIC THRESHOLD IN PATIENTS WITH ME/CFS**									
Hodges et al. (46)	Fukuda, CCC, ICC	10	134	128	104.6	10	133	128	103.8
Keller et al. (28)	Fukuda	22	113	123	91.7	22	108	123	87.7
Vermeulen et al. (65)	Fukuda	15	110	129	85.4	15	112	129	87.0
Sample weighted mean		—	116.5	126.0	92.4	—	114.5	126.0	90.9
95% confidence interval		—	112.8–120.2	125.2–126.9	89.6–95.2	—	110.8–118.4	125.2–126.9	88.1–93.6
**MEASUREMENTS AT VENTILATORY ANAEROBIC THRESHOLD IN CONTROL SUBJECTS**									
Hodges et al. (46)	Fukuda, CCC, ICC	10	137	127	108.1	10	146	127	108.3
Vermeulen et al. (65)	Fukuda	15	111	129	86.2	15	118	129	91.6
Sample weighted mean		—	121.4	128.0	95.0	—	129.2	128.0	101.0
95% confidence interval		—	95.9–146.9	127.4–128.5	88.9–101.0	—	121.4–137.0	127.4–128.5	95.4–107.6

*n, sample size; ME/CFS, myalgic encephalomyelitis/chronic fatigue syndrome; CCC, Canadian Consensus Criteria; ICC, International Consensus Criteria*.

During the first CPET at VAT, individuals with ME/CFS achieved 92.4% of predicted heart rate (95%ConI: 89.6–95.2) and control subjects achieved 95.0% of predicted heart rate (95%ConI: 88.9–101.0), which was not significantly different (*p* = 0.387). However, during the second CPET at VAT, individuals with ME/CFS decreased slightly (90.6%, 95%ConI: 88.1–93.6%) while matched control subjects increased (101.1%, 95%ConI: 94.5–107.6%), resulting in a significant difference in percentage of predicted HR achieved between groups on the second CPET (*p* < 0.01). The observed reduction of 10 beats per minute in patients with ME/CFS compared to matched control subjects in the post-exertional state also appears to be clinically important, because it represents a decrement in repeated submaximal functioning that is consistent with the relatively narrow physiological range for many usual daily activities. The relatively small pooled sample sizes for this analysis suggest the need for future studies to examine test-retest effects in chronotropic and other responses to exercise, in the context of measurements obtained during standardized maximal CPET methodologies. The heterogeneity of findings at VAT on serial CPET also highlights the need to adhere to strict patient selection standards and a uniform methodology for conducting CPET and selecting VAT across future studies (75).

### Comparisons Between Levels of Severity in ME/CFS

One article contained data 179 individuals with ME/CFS that allowed for analysis of chronotropic response based on cardiovascular impairment (Table 6) (27). In this study, subjects were classified according to the American Medical Association Guidelines for the Evaluation of Permanent Impairment (AMA) impairment level based on peak volume of oxygen consumed (VO_2_). Classifications included no impairment (*n* = 20), mild impairment (*n* = 67), moderate impairment (*n* = 72), and severe impairment (*n* = 20). At maximal exertion, individuals with no impairment achieved 91.1% of age-predicted maximum HR. There was a general trend toward a declining percentage of age-predicted maximum HR with increasing AMA impairment level. Individuals with ME/CFS and mild AMA impairment reached 83.1% of age-predicted maximum HR, whereas those with moderate AMA impairment demonstrated 75.1% of age-predicted maximum HR, and individuals with severe AMA impairment only achieved 67.6% of age-predicted maximum HR. These data suggest the potential presence of a clinically important interaction between cardiovascular impairment and CI, in which functional impairment categories could be related to increasing levels of autonomic impairment.

## Relevance of CI to Patho-etiological Studies in ME/CFS

**Figure 4:**
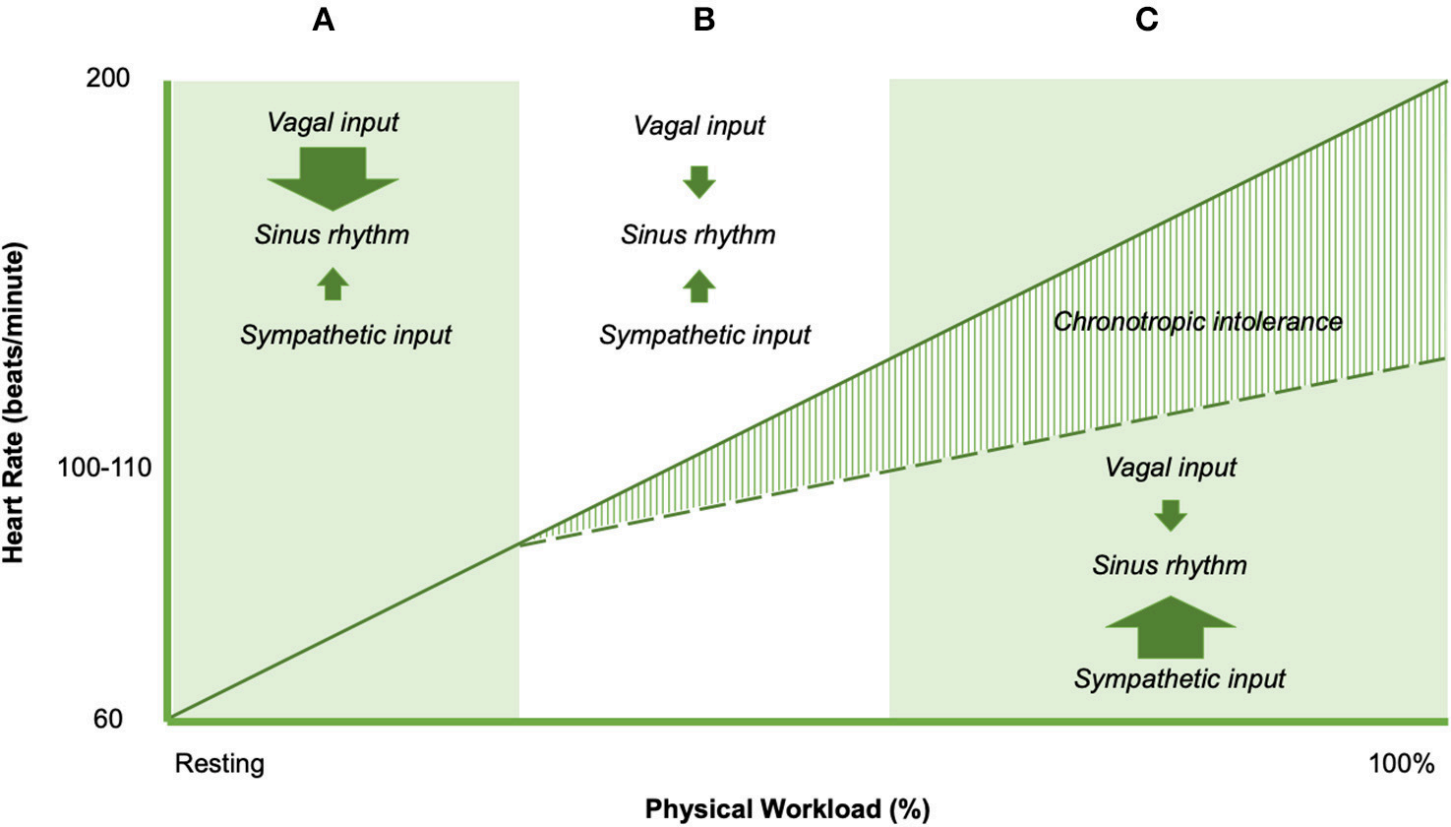
Heart rate responses to exercise in non-ME/CFS (solid line) and ME/CFS (dashed line). Arrow sizes represent the direction and magnitude of the influence of the dominant controllers of heart rate in shaded region.

Chronotropic responses during exercise result from a balance of neural and humoral influences on the intrinsic firing rate of sinoatrial (SA) and atrioventricular (AV) node cells (Figure 4). The normal discharge rate of sinoatrial node cells provides 100 beats per minute (76). In the resting state influence from parasympathetic fibers from the vagus nerve depresses heart rate to the normal range of 60–100 beats per minute. Parasympathetic effects on the SA and AV nodes are mediated through cholinergic inputs (76). Acetylcholine binds to muscarinic receptors on the cardiac muscle, SA node, and AV node (76). Sympatho-adrenal-medullary responses mediate the increase in heart rate commensurate with exercise workload. Sympathetic fibers innervate the myocardium, conduction system, SA node, and AV node, which act on cardiac structures through the release of epinephrine at the neuromuscular junction (76) In addition, cardiac structures are responsive to circulating catecholamines from blood (epinephrine) (76). ß_1_-adrenoreceptors and ß_2_-adrenoreceptors are located on the myocardium, conduction system, SA node, and AV node, which bind epinephrine and norepinephrine (76). The net effect of adrenergic inputs is to increase heart rate above 100 beats per minute, such as during periods of distress or exercise. Following adrenergic/cholinergic binding on cardiac structures, local signal transduction is responsible for observed changes in heart rate (76).

The balance of cardiac neural control necessary for normal exercise-related changes in heart rate implicates the potential importance of impaired cardiac neural control to explain impairments in exercise-related heart rate change (77). Specifically, blunted changes in exercise-related heart rate could be linked to four major abnormalities of cardiac neural regulation. Down-regulation of ß_1_ and/or ß_2_ adrenoreceptors might result in adrenergic insensitivity, and limited rise in heart rate during exercise. Second, sympathetic fiber dysfunction could result in decreased norepinephrine output, which would reduce the adrenergic effects on cardiac structures and reduce exercise-related changes in heart rate. Third, diminished sympatho-adrenal-medullary activation may result in smaller rises in epinephrine. Finally, a relative dominance of vagus (cholinergic) inputs inhibit the influence of epinephrine and norepinephrine on local cardiac structures, and therefore blunt heart rate increases with increasing exercise workloads. This “cholinergic dominance” hypothesis would appear to be in line with existing conceptual work by Van Elzakkar (78). However, the specific mechanisms that cause or predispose to CI largely remain unclear. Intolerance of sympathetic autonomic endocrine signaling, myocardium, SA node, AV node, and conduction system all have been implicated in CI in various pathophysiological conditions (22, 79), and also have been suggested as a cause of PEM in ME/CFS (80, 81).

## Relevance of CI to Exercise Testing and Analeptic Management for ME/CFS

One approach to circumvent potential challenges associated with maximal exercise testing is the use of submaximal exercise testing. Submaximal exercise paradigms have been proposed as a safer alternative to maximal cardiopulmonary exercise testing (82), because it is thought to be less likely to create severe, long-lasting symptoms in people with ME/CFS. One example of a submaximal test paradigm involves a sustained 25-min bout of work at 70% of age-predicted maximum heart rate (83). This type of “submaximal” physiological stressor has been used in a number of studies involving patients with ME/CFS. However, the presence of abnormal heart rate responses to exercise in people with ME/CFS suggests a potential to over-estimate workload based on predicted heart rate, which in turn, risks having subjects exert harder than intended during tests that are putatively “submaximal.”

Although participants with ME/CFS in studies that use submaximal exercise test paradigms generally demonstrate averaged exercise heart rates that are statistically similar to control subjects, it seems notable that participants achieve statistical similarity at significantly lower averaged workloads and averaged VO_2_ (83). Because cardiac, pulmonary, and metabolic measurements using submaximal protocols are not performed to peak exertion, it is impossible to determine the AMA impairment category or evaluate VAT for each subject, which prevents the estimation of potential effects of CI on actual exertion levels for patients with ME/CFS. In addition, it is possible that at least some patients with ME/CFS in studies using submaximal exercise paradigms could have been performing maximal tests. For example, Cook et al. (83) published data on RER values for patients with ME/CFS and controls. The reported 99% confidence interval for averaged respiratory exchange ratio was 1.1 for people with ME/CFS but not control subjects. This observation suggests the potential for maximal exertion in some participants with ME/CFS but not control subjects (83), because RER values >1.15 are one criterion to determine a maximal CPET (84). These data point to important cautions about extrapolating the idea of submaximal tests to people with ME/CFS without individualized measurement and analysis.

Consideration of CI during submaximal exercise is critical to understanding the results of exercise studies using these putatively submaximal methodologies. The presence of CI suggests that it is difficult to determine whether each participant with ME/CFS receives a standardized dose of the physiologic stressor; indeed, the previously observed trend of CI makes it possible that the participants with ME/CFS who have more impairment may have received a proportionally greater stressor than participants with less impairment. For example, individuals classified as having no AMA impairment might be exerting sub-maximally at approximately 70% of age-predicted heart rate but individuals with moderate to severe AMA impairment actually might perform supra-maximally (33). Given the relatively low number of participants with ME/CFS in studies using submaximal exercise methodologies, careful standardization of the exercise stressor appears important to ensure that measures of blood chemistry, imaging and cognitive-perceptual data do not have outliers. Uniformity in sample characteristics and exercise stressor is made more important by the fact that neither sample size calculations nor tests of data normality are commonly reported in studies using submaximal methodologies.

Volume of oxygen consumed (VO_2_) depends on a robust chronotropic response because heart rate rise during exercise increases cardiac output, and therefore the amount of oxygen available to tissues. Thus, CI may explain low achieved VO_2_ at peak and VAT, especially when observed on a second CPET (26). These data suggest an interaction effect between group and test at VAT, in which there is a greater reduction in VO_2_ at VAT in people with ME/CFS than matched, sedentary control subjects (26). We measured a 19.4% difference in VO_2_ at VAT on a second CPET, which we believe reflects a clinically significant reduction in capacity for normal daily activities or ADLs (Table 5).

**Table 5:** Raw and percent differences in metabolic equivalents between individuals with myalgic encephalomyelitis/chronic fatigue syndrome (ME/CFS) and matched sedentary individuals during serial cardiopulmonary exercise testing (CPET), based on re-analysis of data from Snell et al. (26).

	**ME/CFS (*n* = 51)**	**Control (*n* = 10)**
**CPET1**		
VO_2_, Peak	21.51 (4.09) 20.34–22.71	25.04 (4.41) 22.35–27.73
METs, Peak (Calculated)	6.15 (1.17) 5.81–6.49	7.15 (1.26) 6.39–7.92
% Difference, Peak	−16.3%	
VO_2_, VAT	12.74 (2.85) 11.92–13.55	13.83 (2.79) 12.00–15.67
METs, VAT (Calculated)	3.64 (0.81) 3.41–3.87	3.95 (0.78) 3.43–4.48
% Difference, Peak	−8.2%	
**CPET2**		
VO_2_, Peak	20.44 (4.47) 19.25–21.63	23.96 (4.30) 21.27–26.65
METs, Peak (Calculated)	5.84 (1.28) 5.50–6.18	6.85 (1.23) 6.08–7.61
% Difference, Peak	−14.7%	
VO_2_, VAT	11.36 (2.91) 10.39–12.01	14.12 (3.26) 12.29–15.96
METs, VAT (Calculated)	3.25 (0.83) 2.97–3.43	4.03 (.93) 3.51–4.56
% Difference, VAT	−19.4%	

*Decremented performance was noted in individuals with ME/CFS on the second CPET at peak exertion and ventilatory anaerobic threshold, indicating the physiological correlates of post-exertional malaise. Measurements are expressed as mean (standard deviation) and 95% confidence interval. CPET, cardiopulmonary exercise test; HR, heart rate; ME/CFS, myalgic encephalomyelitis/chronic fatigue syndrome; METs, metabolic equivalents; VAT, ventilatory anaerobic threshold*.

**Table 6:** Chronotropic response to exercise measured during a single maximal cardiopulmonary exercise test in individuals with myalgic encephalomyelitis/chronic fatigue syndrome (ME/CFS), based on re-analysis of data from VanNess et al. (27).

	**None (*n* = 20)**	**Mild (*n* = 67)**	**Moderate (*n* = 72)**	**Severe (*n* = 20)**
**PEAK EXERTION (MEASURED HEART RATE)**				
Predicted HR	179	177	177	173
Actual HR	163	147	133	117
% Predicted	91.1	83.1	75.1	67.6
**70% EXERTION (CALCULATED HEART RATE)**				
Predicted HR	126	124	124	121
Actual HR	114	102	93	82
% Predicted	90.4	82.3	75.0	67.8

*(The authors used the Holmes criteria to identify ME/CFS). At both peak exertion and ventilatory anaerobic threshold, the difference between age-predicted heart rate and observed heart rate increased as American Medical Association metabolic impairment category worsened. HR, heart rate*.

Many ADLs are conducted above VAT in people with ME/CFS (Table 7), which may predispose them to the development of PEM. A single bout of exercise may lower the VO_2_ observed on a second test, which causes even more ADLs to exceed VO_2_ at VAT in the post-exertional state. This observation is relevant because energy expenditures at, or close to VAT, represent vigorous activity and can be sustained for only short periods of time (Table 7). The International Labor Organization regard 30% or less of maximal VO_2_ as the threshold for acceptable physiological demands over an 8-h work day. For a 12-h work day this is reduced to 23% or less and limited to physically light work. Extended working hours are not advisable when job-related mental or emotional stresses are high. Estimated energy expenditures for most occupations and life activities can be found in the online Compendium of Physical Activities (85).

**Table 7:** Oxygen needs (expressed in METs) required to complete common activities of daily living (85), and assessment whether they occur under ventilatory anaerobic threshold (VAT) in individuals with myalgic encephalomyelitis/chronic fatigue syndrome (ME/CFS) and sedentary individuals.

**Activity**	**MET requirement (ml/kg/hr)**	**Under VAT?**		
		**ME/CFS**		**Sedentary**
		**Pre PEM**	**Post PEM**	
Circuit training	4.3	No	No	No
Driving automobiles	2.5	Yes	Yes	Yes
Folding laundry	2.3	Yes	Yes	Yes
**Food preparation**	**3.5**	**No**	**No**	**Yes**
Food shopping	2.5	Yes	Yes	Yes
**Light bicycling**	**3.5**	**No**	**No**	**Yes**
**Light calisthenics**	**3.5**	**No**	**No**	**Yes**
Lying quietly	1.0	Yes	Yes	Yes
**Making the bed**	**3.5**	**No**	**No**	**Yes**
Mild stretching	2.3	Yes	Yes	Yes
**Moderate bicycling**	**6.8**	**No**	**No**	**Yes**
**Moderate cleaning**	**3.5**	**No**	**No**	**Yes**
**Playing with children**	**3.5**	**No**	**No**	**Yes**
**Scrubbing floors**	**3.5**	**No**	**No**	**Yes**
Showering	2.0	Yes	Yes	Yes
Sitting quietly	1.3	Yes	Yes	Yes
Sleeping	0.95	Yes	Yes	Yes
Standing quietly	1.3	Yes	Yes	Yes
**Sweeping**	**3.3**	**Yes**	**No**	**Yes**
**Vacuuming**	**3.3**	**Yes**	**No**	**Yes**
Vigorous bicycling	8.8	No	No	No
Walking <2.0 mph	2.0	Yes	Yes	Yes
**Walking 3.0 mph**	**3.5**	**No**	**No**	**Yes**
Walking 3.5 mph	4.3	No	No	No
Washing dishes	2.5	Yes	Yes	Yes
**Washing windows**	**3.5**	**No**	**No**	**Yes**
Watering plants	2.5	Yes	Yes	Yes

*Activities falling under the 95% confidence interval for VAT from data reported by Snell et al. (26) were considered under VAT. Likely differences in activity tolerance between individuals with ME/CFS and sedentary individuals appear in bold. ME/CFS, myalgic encephalomyelitis/chronic fatigue syndrome; METs, metabolic equivalents; PEM, post-exertional malaise; VAT, ventilatory anaerobic threshold*.

## Conclusion

This literature synthesis supports the presence of abnormally blunted HR responses to activity in people with ME/CFS, at both maximal exertion and submaximal VAT. Pathophysiological processes consistent with autonomic dysregulation should be prioritized for etiologic studies in ME/CFS, independent of distal pathogenic causes and proximal multi-system effects. The abnormal heart rate response to exercise in people with ME/CFS indicates that exercise testing based on a percentage of maximal heart rate cannot be considered “submaximal” in people with ME/CFS and presents a clear risk for supramaximal exertion during “submaximal” exercise tasks in the most severely involved individuals. Pacing self-management plans based on age-predicted heart rate thresholds should be viewed with caution, because the chronotropic response is impaired in people with ME/CFS. Threshold heart rates for effective analeptic management and the etiology of observed CI in people with ME/CFS should be formally established through adequately powered studies that involve serial maximal CPET methodologies.

## Author Contributions

All authors listed have made a substantial, direct and intellectual contribution to the work, and approved it for publication.

### Conflict of Interest Statement

The authors declare that the research was conducted in the absence of any commercial or financial relationships that could be construed as a potential conflict of interest.
